# The wear and tear on health: What is the role of occupation?

**DOI:** 10.1002/hec.3563

**Published:** 2017-09-13

**Authors:** Bastian Ravesteijn, Hans van Kippersluis, Eddy van Doorslaer

**Affiliations:** ^1^ Erasmus School of Economics Erasmus University Rotterdam Rotterdam the Netherlands; ^2^ Department of Health Care Policy Harvard Medical School Boston MA USA; ^3^ LIRAES(EA4470) Université Paris Descartes ‐ Sorbonne Paris Cité Paris France; ^4^ Institute of Health Policy and Management Erasmus University Rotterdam Rotterdam the Netherlands; ^5^ Tinbergen Institute Rotterdam the Netherlands

**Keywords:** dynamic models, occupational stressors

## Abstract

Health is well known to show a clear gradient by occupation. Although it may appear evident that occupation can affect health, there are multiple possible sources of selection that can generate a strong association, other than simply a causal effect of occupation on health. We link job characteristics to German panel data spanning 29 years to characterize occupations by their physical and psychosocial burden. Employing a dynamic model to control for factors that simultaneously affect health and selection into occupation, we find that selection into occupation accounts for at least 60% of the association. The effects of occupational characteristics such as physical strain and low job control are negative and increase with age: late‐career exposure to 1 year of high physical strain and low job control is comparable to the average health decline from ageing 16 and 6 months, respectively.

## 1 INTRODUCTION

It is well known that average health and life expectancy display a clear gradient by occupation (e.g., Marmot et al., 1991). Manual workers in the United States, for example, are 50% more likely to die within a given year than workers in managerial, professional, and executive occupations (Cutler, Lleras‐Muney & Vogl, 2008). The mortality rate for manual workers in Europe is higher than for nonmanual workers throughout the age distribution, and this gap has widened over time (Mackenbach et al., 2003). For the Netherlands, Ravesteijn, van Kippersluis, and van Doorslaer (2013) find a strong gradient in self‐assessed health by occupational class—particularly at an older age—and note that 20% of elementary workers 1Elementary occupations consist of simple and routine tasks which mainly require the use of hand‐held tools and often some physical effort.at the age of 60 have exited the workforce into disability, as opposed to 8% of workers in occupations that require academic training.

Although the occupation/health gradient is widely documented, less is known about the underlying mechanisms that generate it. Occupation may exert a causal effect on health, but the strong correlation may also stem from reverse causality, with health constraining occupational choice. Moreover, individuals in different occupational groups can differ in other—observed and unobserved—“third factors” that influence health. For example, manual workers are typically less educated or possess different genetic predispositions than nonmanual workers. Moreover, workers choose their occupation on basis of preferences for health and mortality risks (DeLeire & Levy, 2004). Both reverse causality and third factors may lead to selection effects: People with good health prospects are selected into certain types of occupations. As a result, the magnitude of the association between occupation and health is likely to be (much) higher than the magnitude of the causal effect of occupation on health.

Many studies have documented strong associations between occupational characteristics and health (see e.g., Kunst et al., 1999; Goodman, 1999), but few have attempted to obtain estimates of a causal effect, and those that do often focus on specific occupations or specific types of exposure to unhealthy circumstances. 2For example, Bongers et al. (1990) study back pain among helicopter pilots.The relationship between occupation and health has received surprisingly little attention in the economics literature, but interest in the topic has grown in recent years. Case and Deaton (2005) show that the self‐reported health of manual workers is lower and declines more rapidly with age than that of nonmanual workers. Choo and Denny (2006) report similar patterns for Canadian workers while controlling for a more extensive set of lifestyle factors and suggest that manual work has an independent effect on health over and above any differences in lifestyle across occupations. Using the longitudinal Panel Study of Income Dynamics, Morefield, Ribar, and Ruhm (2012) estimate that 5 years of blue‐collar employment predicts a 4% to 5% increase in the probability of moving from good health to poor health. 3Apart from current occupation, a worker's entire occupational history is likely to affect current health. Thus, Fletcher and Sindelar (2009) use father's occupation during childhood and the proportion of blue‐collar workers in the state as instrumental variables for first occupation and find that a blue‐collar first occupation negatively affects self‐assessed health. Kelly, Dave, Sindelar, and Gallo (2012) question the statistical relevance of the 2 instrumental variables used in Fletcher and Sindelar (2009) and instead propose methods developed by Lewbel (2012) and Altonji, Elder, and Taber (2005) to investigate the causal effect of first occupation on health. They find that entering the labor market as a blue‐collar worker raises the probabilities of obesity and smoking by 4% and 3%, respectively, which indicates that the effect of occupation on health may—at least in part—be transmitted through lifestyles.


The most comprehensive attempt to estimate the health impact of occupation is Fletcher, Sindelar, and Yamaguchi (2011) who combine information on the physical requirements of work and environmental conditions taken from the Dictionary of Occupational Titles with occupational information in the Panel Study of Income Dynamics. Their aim is to estimate the health impact of 5‐year exposure to physical and environmental conditions as the Dictionary of Occupational Titles lacks information on psychosocial stressors. Controlling for first‐observed health and five‐period lagged health in their empirical model, they estimate negative health effects of physical requirements and environmental conditions. They acknowledge that the potential endogeneity of occupation and occupational change does not allow for a causal interpretation of their random effects estimates. Their data also do not permit to disentangle the contributions of physical and psychosocial occupational stressors.

In sum, the literature so far has failed to establish to what extent the strong association between occupation and health reflects a causal effect of occupation on health and to what extent it reflects the selection of unhealthy individuals into occupations with harmful job characteristics. In part, this is due to the difficulty of finding credible sources of exogenous variation in occupation. Previous studies have also had only limited success in disentangling the health effects of different types of occupational stressors.

Our contribution to the literature is threefold. First, we derive an empirical specification that is grounded in a theoretical model of occupation and health over the life cycle. The explicit link between the theory and the empirical specification (a) identifies the sources of health‐related selection into occupation, (b) shows how our econometric estimators relate to the structural parameters, and (3) details the conditions under which our dynamic panel data estimates allow for a causal interpretation. These insights provide a theoretical foundation for the dynamic panel data estimation.

Second, we estimate an empirical model, on German longitudinal data, that can account for various sources of selection: (a) unobserved time‐invariant variables due to the inclusion of individual fixed effects, (b) time‐varying observed variables such as age and wave dummies, and (c) time‐varying unobserved shocks that exponentially die out through the inclusion of the lagged dependent variable. We argue that with panel data spanning 29 years and in the absence of credible sources of exogenous variation in occupation, our model provides the most promising attempt to disentangle health‐related selection into occupation from the effects of occupation on health.

Third, we show that blue‐collar occupations are both more physically demanding and more often characterized by low job control. Previous studies have often characterized occupation with a binary indicator of manual versus nonmanual occupation or have focused only on the physical aspects of occupation. This approach has left the contributions of the various ergonomic and psychosocial stressors unseparated and made clear policy conclusions difficult to draw. By linking German data on occupational stressors to individual‐level longitudinal data, we are able to unravel the health effects of job characteristics in greater detail.

Our findings suggest that at least 60% of the association between physical demands at work and self‐reported health stems from the selection of individuals with worse health (prospects) into occupations with high physical demands and that the same holds for the degree of job control. Although we do not directly observe the correlates of health responsible for the selection of individuals into different occupation, the literature suggests that factors such as overweight (Harris, 2015), gender, and risk preferences (Dohmen & Falk, 2011) play an important role. Our findings contribute to the literature on occupational sorting (Keane & Wolpin, 1997; DeLeire & Levy, 2004; Lee, 2005; Dohmen & Falk, 2011) by quantifying the contribution of health‐related sorting into occupations in the association between occupation and health.

Under the admittedly stringent assumptions laid out in the theoretical framework, we estimate that the average effect of 1 year exposure to a one standard deviation increase in the degree of physical strain (e.g., working as a toolmaker instead of as a teacher) is comparable to the effect of ageing 9 months, and the effect increases with age. A lower degree of control over daily activities at work (e.g., a secretary versus a librarian) is harmful to health at older ages but not at younger ages. We estimate that exposure to a one standard deviation increase in handling heavy burdens between the ages of 50 and 54 leads to a health deterioration that is comparable to ageing 16 months or 130% of the yearly biological ageing rate. The estimated effect of exposure to low job control between the ages of 50 and 54 is comparable to ageing 6 months or 50% of the yearly biological ageing rate.

## 2 OCCUPATION AND HEALTH OVER THE LIFE CYCLE

In the economics literature, health is treated as a durable capital stock that depreciates with age and can be increased with investment (Grossman, 1972). The age‐related health depreciation rate is exogenous, but an individual can invest in his health by purchasing preventive and curative medical care. The effect of behavior on health can be positive or negative. Occupational choice can be understood as a form of health disinvestment/erosion: An individual chooses an occupation that is characterized by a set of potentially harmful occupational stressors (Case & Deaton, 2005; Galama & van Kippersluis, 2015). Occupations with more harmful characteristics may yield higher earnings than other less harmful occupations in the choice set of the individual, which is known as the compensating wage differential (Smith, 1974; Viscusi, 1978; DeLeire and Levy, 2004; DeLeire, Khan & Timmins, 2013; Guardado and Ziebarth, 2013). The additional earnings may be used to partially offset the detrimental effect of work on health by investing in health or to increase consumption. This economic paradigm is useful for distinguishing between the sources of health‐related selection into occupation.

Our empirical investigation is based on a theoretical model of an individual maximising the expected present value of lifetime utility, which is derived from consumption *c* and health *h*, by choosing levels of consumption *c*, occupational stressors in vector **o**, and health investment *m*. Each occupation is characterized by physical and psychosocial occupational stressors that tend to be clustered, that is, occupations with low psychosocial workload are often characterized by high physical demands. Future utility is discounted at discount rate *β*. The information set I includes endowments *e* and permanent health *h*_*p*_, all state and choice variables up to time *t*, and all future values of the ageing rate, but not future unanticipated health shocks *η*.

(1) $$max_{{\left\{c_{t+j},o_{t+j},m_{t+j}\right\}}_{j=0}^{T-t}}E\left[\sum_{j=0}^{T-t}\beta^{\;j}u(c_{t+j},h_{t+j})|I_{t}\right]$$

The health production function depends on (a) characteristics and circumstances that remain constant over time that are embodied by permanent health *h*_*p*_=*f*(*e*), which is a function of endowments and reflects all circumstances and personal characteristics that remain constant over the life cycle; (b) anticipated health deterioration due to ageing *a*; (c) a vector of (physical and psychosocial) occupational characteristics **o**; 4In Section 3, we will link this to the seminal work by Karasek (1979) on occupational stressors.(d) medical investment *m*; and (e) exogenous health shocks *η*. The effect of occupational characteristics on health, ***γ***_*o*_, is nonpositive, and 0≤*θ*≤1 reflects diminishing marginal benefits to health investment. The effects of occupational stressors, health investments, and shocks are assumed to decay at the same rate *ϕ*, which lies between 0 and 1. Total lifetime T is exogenous and known to the individual.

(2) $$h_{t+j}=h_{p}+\sum_{k=2}^{t+j}\left(a_{k}+\phi^{t+j-k}(\gamma_{o}^{\prime }o_{k-1}+\gamma_{m}m_{k-1}^{\theta }+\eta_{k})\right)$$

Expenditures on consumption and health investment, at prices *p*_*c*_ and *p*_*m*_, respectively, should not exceed the net value of wage earnings. The individual can lend and borrow at real interest rate *r*, but he must repay any remaining debt at the end of his life. Wage *w* is a function of (a) current occupational choice **o**, (b) current health *h*, and (c) endowments *e*.

(3) $$s.t.\sum_{k=1}^{T}(p_{c}c_{k}+p_{m}m_{k})\leq \sum_{k=1}^{T}{\left(1+r\right)}^{k-1}w(o_{k},h_{k};e)$$

Consumption, health investment, and occupational choice are chosen by equating marginal benefit with marginal cost. The marginal utility of consumption is equal to the shadow price of income *λ* multiplied by the price of consumption.

(4) $$\frac{\partial u_{t}}{\partial c_{t}}=\lambda p_{c}$$

For each occupational attribute *o*_*l*_ in vector **o**, the marginal benefit of occupational stress is represented by the product of *λ* and the instantaneous wage premium. The marginal cost includes the marginal deterioration of health in all future periods multiplied by (a) the discounted marginal utility of future health and (b) the product of *λ* and the present value of the marginal wage returns to future health.

(5) $$\lambda \frac{\partial w_{t}}{\partial o_{t,l}}=-\sum_{j=1}^{T-t-1}\frac{\partial h_{t+j}}{\partial o_{tl}}\left[\beta^{j}\frac{\partial u_{t+j}}{\partial h_{t+j}}+\lambda {\left(\frac{1}{1+r}\right)}^{j}\frac{\partial w_{t+j}}{\partial h_{t+j}}\right]\;\forall l$$

Health investment is the “mirror image” of occupational choice. The marginal benefit (the product of the marginal effect of health investment on health and both the discounted marginal utility of health and the marginal wage returns to health in all future periods) is equated with marginal cost (the product of the shadow price of income and the price of medical care).

(6) $$\sum_{j=1}^{T-t-1}\frac{\partial h_{t+j}}{\partial m_{t}}\left[\beta^{j}\frac{\partial u_{t+j}}{\partial h_{t+j}}+\lambda {\left(\frac{1}{1+r}\right)}^{j}\frac{\partial w_{t+j}}{\partial h_{t+j}}\right]=\lambda p_{m}$$

The theoretical framework shows how an individual takes the future consequences of his decisions into account while deciding on the optimal levels of harmful occupational stressors. Three insights from the theory are particularly noteworthy. First, both time‐invariant initial endowments *e*—in the form of, for example, physical ability, intelligence or taste for adventure—and time‐varying factors such as health shocks *η*—for example, a car accident or the onset of a disease—may influence both occupational choice and health status through (a) the marginal utility of health, (b) the marginal wage returns to health, and (c) the shadow price of income *λ*. This finding indicates that workers may select themselves into certain types of occupations depending on exogenous factors that directly influence health. Observed health differences across occupational classes should therefore not be interpreted as evidence of a causal effect of occupation on health.

Second, health‐related selection into occupation is not only exogenously determined by endowments and shocks: Individuals choose their levels of health investment. Health investment may be correlated with occupational choice because (a) exogenous factors influence both health and occupational choice and (b) workers may choose to offset occupation‐related health damage by investing in health (e.g., a bricklayer may seek physiotherapeutic treatment for his back pain or a manager may take yoga classes to help handle psychological stress).

Third, the relationship between work and health may change over the life cycle for three reasons. First, the health production function (6) illustrates that the expected wage returns on health investment decrease as the individual approaches retirement age, which implies that individuals have fewer incentives to offset occupational damage to health by medical investment. 5However, a model that endogenizes length of life as a function of health can explain an increase in medical investment at older ages.Second, ***γ***_**o**_ may change over the lifetime, for example, if health at older ages is more susceptible to wear and tear at the workplace. Third, the marginal effect of health repair may decrease with age to such an extent that full health repair is no longer feasible at older ages. 6Our model does not incorporate real‐world labor market rigidities, but such rigidities may also prevent individuals from switching occupations at older ages to optimize their exposure to occupational stressors.


In sum, the theory imposes the following conditions on our empirical identification strategy: It should (a) account for factors that can influence selection into type of occupation and may also be related to health, (b) allow for behavioral adjustments that affect health may coincide with occupational choice, and (c) accommodate the changing relationship between occupation and health over the life cycle. This requires individual‐level data that includes information on health, observes occupational stressors for working individuals, and repeated measures of these variables over an extensive period of time.

## 3 THE GERMAN SOCIOECONOMIC PANEL

**Figure 1:**
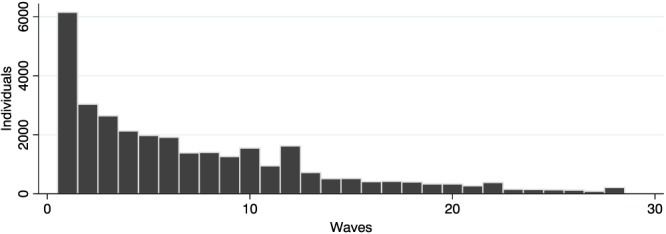
Number of individuals by number of observed waves. Note: Each bar shows the number of individuals by the total number of (not necessarily consecutive) observed waves in which the individual was employed in the previous period and between 16 and 65 years old. Source: SOEP v29 [Colour figure can be viewed at wileyonlinelibrary.com]

The German Socioeconomic Panel (SOEP) is a representative longitudinal household survey that started in 1984. We use data from the 29 subsequent annual waves. Respondents are followed over multiple waves, but the panel is unbalanced as many respondents enter the sample after 1984 or leave the sample before 2012. The sample is restricted to 222,726 person‐wave observations for which we observe occupation in the previous year, educational attainment, and health in the previous and in the current year. It includes individuals between 16 and 65 years old who were employed in the previous period. Sample sizes per wave range between 4,702 in 1989 and 11,798 in 2003. Figure 1 shows that we observe 31,216 individuals for at least one period and 10,577 individuals for at least nine periods, a more restricted sample that we will use in a robustness check.

### 3.1 Health

The SOEP has three general health measures: We use health satisfaction (HSAT), measured on an integer scale from 0 to 10, as our main outcome variable. Self‐assessed health (SAH), measured on a 5‐point scale ranging from bad to very good, and SF12, a physical and mental health summary score on basis of 12 survey questions, are used for robustness checks because they were only included since 1992 (SAH) and 2002 (SF12).

### 3.2 Occupation

Occupational titles are reported according to the International Standard Classification of Occupations of the Organisation for Economic Co‐operation and Development (OECD; ISCO‐88). 7See http://www.ilo.org/public/english/bureau/stat/isco/isco88/ for more information.This gives us 307 occupational titles that were grouped into nine major occupational groups ranked by the OECD classifications. White‐collar workers include legislators, senior officials, managers, professionals, technicians, associate professionals, and clerks. We define blue‐collar workers as service workers and shop and market sales workers, skilled agricultural and fishery workers, craft and related‐trades workers, plant and machine operators, assemblers, and workers in elementary occupations. These definitions are consistent with the distinction between manual and nonmanual work of Case and Deaton (2005), but the blue‐/white‐collar terminology better reflects the fact that these occupations differ both in terms of physical strain and psychosocial demands. This classification gives a total of 119,456 person‐wave observations for white‐collar occupations and 103,270 observations for blue‐collar occupations.

**Figure 2:**
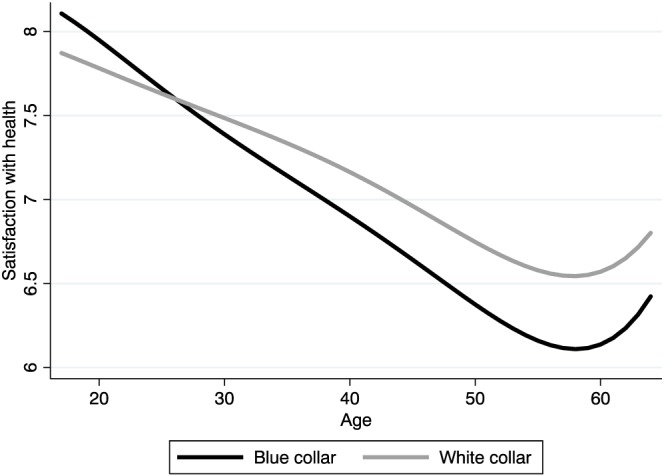
Health for blue‐ and white‐collar workers. Predicted satisfaction with health for blue‐ and white‐collar workers over the life cycle. Those who never worked were dropped. For each of the two groups, we used all observations including years in which an individual did not report an occupation and regressed health on single‐year age dummies and plotted predicted health. Source: SOEP v29 [Colour figure can be viewed at wileyonlinelibrary.com

Figure 2 graphs age‐predicted HSAT for blue‐ and white‐collar workers, where the groups were defined on the basis of whether an individual was in a blue‐ or white‐collar occupation for at least 50% of the waves. On average, blue‐collar workers report better health at younger ages, whereas the opposite is true after the age of 24. HSAT decreases for both blue‐ and white‐collar workers over most of the age range but slightly increases after the age of 57, possibly reflecting selective mortality or stability of health after a certain age. Consistent with Case and Deaton (2005), we find that predicted health decline associated with age in the pooled sample is much stronger among blue‐ than white‐collar workers.

In line with Figure 2, Panel A of Table 1 shows that on average blue‐collar workers report worse health (6.86) than white‐collar workers (7.01). 8Health worsens from the top to the bottom of the OECD occupational ladder: 23% of legislators, senior officials, and managers rate their health with a 5 or less, as opposed to 31% of elementary workers, and 49% of legislators, senior officials and managers rate their health with at least an 8, as opposed to 42% of elementary workers. This pattern is monotonic across the nine ranked major OECD occupational groups.Blue‐collar workers are slightly younger and less likely to be female and have an average of 3 years of schooling less than white‐collar workers.

**Table 1:** Summary statistics for the German Socioeconomic Panel

	HSAT	Age	Female	Schooling	Observations
A. Baseline sample					
All workers	6.94	41.39	.45	12.10	222,726
	(2.05)	(11.47)		(2.71)	
White collar	7.01	42.51	.51	13.34	119,456
	(1.99)	(11.07)		(2.83)	
Blue collar	6.86	40.10	.35	10.66	103,270
	(2.11)	(11.78)		(1.65)	
B. Individuals who were employed in at least nine annual waves					
All workers	6.92	42.16	.43	12.14	151,752
	(2.00)	(10.45)		(2.70)	
White collar	6.98	43.07	.51	13.34	68,585
	(1.96)	(10.12)		(2.82)	
Blue collar	6.85	41.05	.33	10.68	83,167
	(2.05)	(10.74)		(1.61)	

*Notes*. Health satisfaction (HSAT), age, female proportion, years of schooling and monthly labor earnings in the German Socioeconomic Panel. Each wave is viewed as a separate observation. Standard deviations are in parentheses. Source: SOEP v29

Panel B of Table 1 shows descriptive statistics for a restricted sample that we will use in a robustness check. The average age in the restricted sample of individuals who were observed in at least nine waves is approximately 1 year higher than in the full sample. Average health and the proportion of women are slightly lower. The full and restricted samples are similar in terms of education and (blue‐collar) employment.

### 3.3 Occupational stressors

Although the distinction between blue‐ and white‐collar occupations helps us to characterize health differences across broad occupational groups, it does not allow us to identify which occupational stressors associated with blue‐collar occupations matter the most. In addition to *physical strain*, we follow the seminal work by Karasek (1979) and distinguish between two psychosocial stressors that could affect health. First, *job control* is defined by Karasek as decision authority and intellectual discretion: the individual's potential control over his tasks and his conduct during the working day. Second, *psychosocial workload* refers to the degree to which someone is required to work very fast, hard, or to accomplish large amounts of work and whether he or she is short of time.

We have linked our 307 different ISCO 88 occupational titles in the SOEP to information on occupational stressors from the 2005–2006 German Qualification and Career Survey (GQCS). 9Five hundred twenty person‐year observations were dropped from the sample because 15 of the total of 307 occupational titles in the SOEP could not be linked to the GQCS.We use only one wave of the survey to obtain the exposure variation between jobs, since the measurements across waves are not consistent. Hence, we assume a stable mapping of occupational titles to the associated job exposures over the entire observation period, and the variation in occupation solely derives from individuals switching occupations but not from changes in exposures within the same occupation over time.

In the GQCS, individuals are asked about exposure to occupational stressors and can answer with “frequently,” “sometimes,” “rarely,” or “never” (Hall et al., 2010). We compute the proportion of individuals that is exposed to the given occupational stressor and use this exposure score as independent variable in our main analyses. We take the cutoff value closest to the median for each of the occupational stressors, but varying the cutoff values did not alter our results. Each occupational title is assigned three measures of occupational stress, and each of these measures is constructed on the basis of one single variable to allow for an intuitive interpretation of its coefficient. Robustness checks with composite measures of occupational stressors yield similar results (see Section 5.4).

First, physical strain is measured by the degree of heavy lifting that is required for a job. This variable is constructed as an exposure score: the percentage of individuals in each of the 307 occupations who reported sometimes or frequently carrying heavy stocks of more than 20 kg (men) or 10 kg (women) in the GQCS. Thirty‐five percent of all individuals surveyed in the GQCS report that they sometimes or frequently carry heavy stocks. Subsequently, each working individual in the SOEP was assigned the exposure score in the GQCS that pertains to his current occupation. Second, we measure job control as the percentage of workers in a given occupation who reported in GQCS that their work was frequently or sometimes stipulated in the minutest details. Forty‐six percent of respondents reported that this was frequently or sometimes the case. Third, we measure psychosocial workload as the percentage of respondents in a particular occupation that frequently work under great deadline pressure, which is what 56% of all respondents in the GQCS reported.

Although, in the analysis, we use the mapping of occupational stressors into *all* 307 individual job titles, for illustrative purposes, Table 2 shows the exposure of the nine major OECD occupational groups in the SOEP to the three occupational stressors in the GQCS. Two important observations can be made. First, blue‐collar occupations are characterized not only by higher physical strain compared with white‐collar occupations but also by lower job control. This illustrates the importance of disentangling the health effects of separate occupational stressors. Psychosocial workload is somewhat higher for white‐collar occupations.

**Table 2:** Occupational stressors across the major International Standard Classification of Occupations occupational groups

	High	Low job	High	Obser‐
	physical	control	psychosocial	vations
	strain		workload	
Legislators, senior officials, and managers	30	28	69	15,263
Professionals	13	29	62	40,079
Technicians and associate professionals	31	46	57	55,650
Clerks	19	56	51	30,819
Service workers and shop/market sales workers	47	50	44	28,269
Skilled agricultural and fishery workers	79	44	44	3,523
Craft and related workers	65	59	60	46,960
Plant and machine operators and assemblers	54	65	52	22,892
Elementary occupations	55	49	36	14,439

*Notes*. The numbers reflect average percentages for exposure to high physical strain, low job control, and high psychosocial workload aggregated by major ISCO 88 occupational group, based on measures of occupational stress for each of the 307 observed ISCO 88 occupational codes. The number of observations refers to the number of person‐wave observations in our sample and standard errors are reported in parentheses. White‐collar occupations are above the dashed line, and blue‐collar occupations are below the dashed line. Source: SOEP v29, GQCS.

Second, there is ample variation in occupational characteristics even within the major occupational groups. Even though blue‐collar workers are generally more likely to work under more demanding ergonomic conditions and have lower job control compared with their white‐collar counterparts, this may not necessarily be the case for many specific occupations. A simple division into blue‐ or white‐collar occupations therefore hides the considerable heterogeneity within these groups and the clustering of occupational stressors. We will first look at the blue‐collar/white‐collar distinction before investigating the effects of the three occupational stressors on health.

## 4 ESTIMATION OF THE EFFECT OF OCCUPATIONAL STRESSORS ON HEALTH

### 4.1 Model specification

We aim to estimate the structural parameter ***γ***_**o**_ in Equation 2, which refers to the health effects of exposure to occupational stressors **o** in the previous year. Note that the one‐period lag of the health production function (Equation 2), which includes permanent health *h*_*p*_, the health effects of ageing *a*, health investment *m*, and shocks *η*, is

(7) $$h_{t+j-1}=h_{p}+\sum_{k=2}^{t+j-1}\left(a_{k}+\phi^{t+j-1-k}(\gamma_{o}^{\prime }o_{k-1}+\gamma_{m}m_{k-1}^{\theta }+\eta_{k})\right).$$

Substituting Equation 7 into Equation 2, we obtain

(8) $$\begin{array}{ccc} h_{t+j}=(1-\phi )\left(h_{p}+\sum_{k=1}^{t+j-1}\left(a_{k}\right)\right) & +a_{t+j}+\gamma_{o}^{\prime }o_{t+j-1} & \\ & +\gamma_{m}m_{t+j-1}^{\theta }+\phi h_{t+j-1}+\eta_{t+j}. \end{array}$$

Switching to individual notation and demeaning the covariates to eliminate the time‐invariant factors, we obtain a fixed effects within estimator:

(9) $$h_{i,t+j}-{\bar{h}}_{i}=\phi (h_{i,t+j-1}-{\bar{h}}_{i})+\gamma_{o}^{\prime }(o_{i,t+j-1}-{\bar{o}}_{i})+\delta^{\prime }(x_{i,t+j}-{\bar{x}}_{i})+\varepsilon_{i,t+j}.$$

This specification controls for various sources of selection. First, any unobserved heterogeneity that is constant over time and may be correlated with occupation (such as permanent health *h*_*p*_ in Equation 2) is eliminated: $(1-\phi )h_{p}-(1-\phi ){\bar{h}}_{p}=0$. Moreover, we go beyond the traditional fixed effects estimator by controlling for lagged health. As detailed in the theoretical framework, this purges the occupational effect from all time‐varying unobserved shocks, to the extent that the impact of these unobserved shocks decays exponentially at the same rate over time (see Equation 2). The coefficient *ϕ* of the demeaned one‐period lag of health can be interpreted as the decay parameter through which occupational choice **o**, health investment *m*, unanticipated shocks *η* in period *t*−2, and earlier periods affect current health.

**x** is a vector of control variables consisting of age and wave dummies to control for age effects and a common time trend. A less flexible linear approximation of the age effect would bias our estimates of ***γ***_**o**_ if health deteriorates more rapidly at older ages or if workers at older ages would be more or less likely to be exposed to certain occupational stressors.

The error term is $\varepsilon_{i,t+j}=\gamma_{m}(m_{i,t+j-1}^{\theta }-{\bar{m}}_{i}^{\theta })+\eta_{i,t+j}-{\bar{\eta }}_{i}$, which implies two things. First, the ordinary least squares estimator of the coefficient of the lagged dependent variable *ϕ* is biased because regressor $h_{i,t+j}-{\bar{h}}_{i}$ is correlated with part of the error term $\eta_{i,t+j}-{\bar{\eta }}_{i}$. Importantly, however, the estimator is consistent for large T (Nickell, 1981; Bond, 2002). Plausibly, the 29 waves of the SOEP panel satisfy this criterion, and we check the robustness of our results to restricting the sample to individuals who were observed in nine or more waves.

Second, our estimates should be interpreted as the net effect of occupational stressors, including health investment responses to occupational choice. This net effect is the sum of ***γ***_**o**_ and an additional term resulting from a possible correlation between occupational choice and unobserved contemporaneous health investment. Although this may seem restrictive, we argue that this is a relevant parameter of interest for policymakers because it captures both the direct effects of occupation and the indirect effects through health investment responses to occupation.

### 4.2 Sample selection and estimation

Our sample selects individuals who were working in the previous year, since only for them we observe their occupation. Obviously, our results should therefore be interpreted as the treatment effect on the working population—and not on children, students, the unemployed, the disabled, and retirees—but that seems to be the policy‐relevant effect. Note that even for individuals who quit their job in the current period, we can still estimate the effect of occupation in the previous period on their current health.

We use HSAT (on a 0–10 integer scale) as a proxy for health as dependent variable in a linear dynamic panel data model. Ferrer‐i Carbonell and Frijters (2004) and Frijters, Haisken‐DeNew, and Shields (2005) show that for the variable that measures satisfaction with life on a 10‐point scale, assuming ordinality or cardinality makes little difference, such that a linear specification is acceptable. Reporting heterogeneity arising from different subgroups reporting the same objective health status differently (Lindeboom & van Doorslaer, 2004) is eliminated by the individual fixed effect and the age controls, to the extent that reporting heterogeneity is time‐invariant or changes with age. We cannot rule out however that reporting heterogeneity varies with occupation within individuals conditional on age.

### 4.3 Identifying variation

Our fixed effects estimates are based on all within‐individual deviations of occupational stressors and health from their observed averages. Since the occupational stressors, as derived from the GQCS, are assumed constant over time for a given occupational title in the SOEP, the variation in occupational stressors derives solely from individuals switching between occupations over our observation period. Since the variation in occupational stressors is at the level of the 307 occupational titles in the SOEP, we cluster standard errors at that level. Within our sample, 13,150 out of 22,526 individuals have switched occupations, for a total of 31,727 times. The number of upward and downward switches in terms of physical and psychosocial demands balance out, and a large proportion of older workers are exposed to high occupational stress. 10Twenty‐two percent of individuals working in two subsequent years in their early 20s experienced a year‐to‐year job switch that resulted in changes in the level of each of the three occupational stressors. Job switches that result in changes in stressors peak at 24% between the age of 25 and 26, and year‐to‐year switches in occupational stressors still amount to 14% of all workers who are in their early 60s. For physical strain, there are slightly more year‐to‐year switches to lower levels (8.96%) than to higher levels (8.55%). 8.90% of switchers move to an occupation with higher job control while 8.63% switch to an occupation with lower control. Slightly more workers switch to occupations with a higher workload (8.94%) than with a lower workload (8.57%). These small differences are stable across all ages. A substantial proportion of workers in their early 60s are exposed to above‐median levels of the occupational stressors: 40% for physical strain, 37% for low job control, 55% for high workload.


Our fixed‐effects estimator including a lagged dependent variable accounts for all time‐invariant and some time‐varying health‐related reasons (e.g., the lasting effects of health shocks and age) why individuals may switch between occupations. Comparing the ordinary least squares with our preferred fixed‐effects estimator including a lagged dependent variable estimates therefore provides a useful assessment of the importance of health‐related selection into occupation for the association between occupation and health. Under the stringent assumption that after conditioning on individual fixed effects, a lagged dependent variable, and some time‐varying characteristics, all switches between occupations are random, then our estimates reflect the causal effect of occupational stressors on health. In practice, we cannot rule out that unobserved time‐varying shocks (e.g., divorce or an accident) that are not sufficiently captured by lagged health, simultaneously affect occupational switches and health. This would lead to a bias in the estimated effects of occupation, and so even though we employ the term “effects” in the remainder, our estimates cannot literally be interpreted as the causal effect of occupational stressors on health. More plausibly, we estimate an upper bound on the causal effect of occupation on health and a lower bound on the selection effect. 11Although we cannot rule out that some time‐varying unobserved factors operate in the opposite direction, the sign of the bias when excluding fixed effects or the lagged dependent variable renders the case that the true selection effect is larger more plausible.


## 5 RESULTS

### 5.1 Main results

Table 3 shows the main results for six different models, where we first present results for a dichotomous indicator for blue‐collar/white‐collar occupations (columns 1 to 3) and then for occupation as characterized by three occupational stressors (columns 4 to 6). To understand the order of magnitude of the coefficients, note that the average health deterioration of growing 1 year older (obtained from an individual fixed effects regression of satisfaction with health on age) is ‐.0616 (.0008) in our sample.

**Health effects of blue‐collar work.** The bivariate association in column 1 between satisfaction with health and blue‐ or white‐collar occupation in the previous year confirms that blue‐collar workers are in worse health and that the size of this health gap is comparable to the average health effect of ageing 29 months, which is a sizable and economically meaningful difference. Column 2 shows the results for the model described by Equation 9. Much of the association appears to be driven by health‐related selection into blue‐collar occupations because the estimate of the effect is ‐.0493 (.0165) compared with ‐.1480 (.0420) in column 1. When taken at face value, the health effect of exposure to a blue‐collar occupation in the previous year is comparable to the average health effect of ageing 9 months.
  We add an interaction between age and blue‐collar work in column 3 to investigate whether the effect of blue‐collar employment differs with age. The coefficient in the first row of column 3 refers to the hypothetical effect of blue‐collar employment at the age of zero. The coefficients of the interaction term in the second row indicate that blue‐collar employment is harmful to health and that this effect increases with age.
**Health effects of occupational stressors.** Column 4 breaks down occupation into three dimensions of occupational stressors: physical strain, job control, and psychosocial workload in the preceding year. As expected, physical strain and low job control are associated with worse health, whereas psychosocial workload is positively associated with health.
  From our theoretical model, we expect health‐related selection into occupation to partially drive these associations. Column 5 therefore shows estimates of the effects of these three occupational stressors according to the specification in Equation 9, which controls for selection into occupation on the basis of time‐invariant and time‐varying factors. The results imply that at least 61% of the negative association between physical strain and health can be explained by selection. Our point estimate (‐.0826) suggests that a one standard deviation increase in the distribution of physical strain (e.g., being employed as a toolmaker instead of a teacher) leads to a next‐year health deterioration that is comparable to ageing 16 months. Similarly, the coefficient of job control drops by 60% from columns 4 to 5 and is no longer significant, while the estimated effect of the psychosocial workload in column 5 is close to zero, suggesting that the positive association with health may even be entirely driven by selection.
  Column 6 shows that the effects on health of low job control and handling heavy burdens vary with age. The predicted health deterioration due to a one‐standard‐deviation (.26) increase in handling heavy burdens is equal to 0 at age 32. At the age of 50 the point estimate of the effect of moving up the distribution of physical strain by one standard deviation is comparable to ageing 12 months: .26(.4166−.0130×50)=−.0607. Low job control has a negative effect after age 37: Being in a job with a one standard deviation lower job control (e.g., from librarian to secretary) at age 50 leads to a predicted health deterioration comparable to the effect of ageing 4 months: .17(.3741−.0100×50)=−.0214. We conclude that the effects of physical strain and job control are age‐dependent. The coefficient of the interaction between psychosocial workload and age is not significantly different from zero, possibly because workload is only important for certain personality types or for a subset of occupations.

**Table 3:** Estimates of the effect of occupational stressors on health

	(1)	(2)	(3)	(4)	(5)	(6)
Blue collar at t‐1	‐.1480*** (.0420)	‐.0493*** (.0165)	.1662*** (.0593)			
Age × Blue collar at t‐1			‐.0056*** (.0015)			
Physical strain at t‐1				‐.2102*** (.0686)	‐.0826*** (.0279)	.4166*** (.1121)
Low job control at t‐1				‐.0579 (.1011)	‐.0232 (.0398)	.3741** (.1583)
Psychosocial workload at t‐1				.5978*** (.1671)	.0746* (.0413)	‐.1185 (.1642)
Age × Physical strain at t‐1						‐.0130*** (.0029)
Age × Low job control at t‐1						‐.0100** (.0040)
Age × Psychosocial workload at t‐1						.0013 (.0040)
Health at t‐1		.1120*** (.0165)	.1118*** (.0039)		.1120*** (.0039)	.1116*** (.0039)
Individual FE, age and wave dummies	✗	✓	✓	✗	✓	✓
Observations	222,726	222,726	222,726	222,726	222,726	222,726
*R* ^2^	.0013	.5631	.5632	.0026	.5631	.5633

*Notes*. Main results for satisfaction with health as dependent variable. Columns 1–3 use the dummy blue collar as variable of interest (reference category working in a white‐collar occupation), while columns 4–6 use the three occupational stressors as variables of interest. Columns 1 and 4 present an ordinary least squares specification, columns 2 and 5 present a specification with fixed effects (FE, deviation from means) and a lagged dependent variable, and columns 3 and 6 present a FE and lagged dependent variable specification interacting the variable of interest with age. Standard errors clustered at the occupational title level. * indicates significance at the 10% level, ** at the 5% level, and *** at the 1% level. Intercepts not shown.

### 5.2 Cumulative effects

Cumulative health effects can be obtained from the estimated coefficient of the lagged dependent variable *ϕ* in Equation 9. By assumption, *ϕ* is the uniform exponential decay rate at which past health investment, occupational stressors, and shocks affect current health in Equation 2. The point estimates of *ϕ* in Table 3 suggest that roughly 11% of the occupation‐related health deterioration in period *t*−2 persists in period *t*. Using the point estimates in column 6 of Table 3, the point estimate of health deterioration at the age of 55 caused by a one‐standard‐deviation increase in the physical strain between ages 50 to 54 is $\sum_{k=50}^{54}.1116^{54-k}.26(.4166-.0130\times k)=-.0830$, which is comparable to the average health effect of ageing nearly 16 months. Likewise, the point estimate of the effect of working in occupations with a one‐standar‐deviation lower degree of job control between the ages of 50 and 54 is ‐.0315, which is comparable to the effects of ageing 6 months.

### 5.3 Vulnerability to occupational stressors over the life cycle and by gender

**Figure 3:**
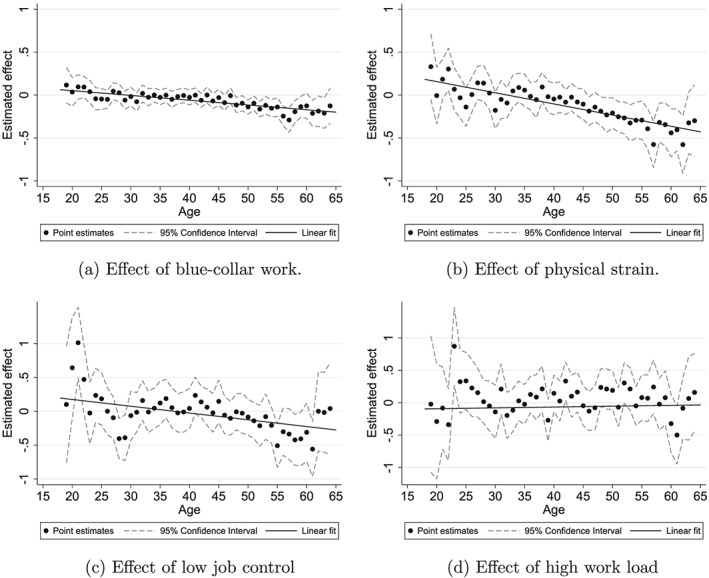
The effects of occupational stressors over the life cycle. Notes: Panel 3a refers to the coefficient of a binary variable indicating blue‐ versus white‐collar occupation interacted with age; panels 3b, 3c, and 3d refer to the estimated health effects of occupations with 100% instead of 0% exposure to each respective stressor. The plotted line shows the linear interaction, corresponding to model 3 in Table 3; the dots and 95% confidence intervals follow from a model with 1‐year age dummy interaction terms instead of the linear interaction term. Source: SOEP v29, GQCS

Health effects may be nonlinear in age. Figure 3 shows the results of regression models that are similar to the specifications of columns 3 and 6 in Table 3 but now include interactions between the occupational variables and single‐year age dummies. In each graph, we additionally plot the interaction term between a linear age term and the occupational measure. Most importantly, the figure indicates that the health effects of the occupational measures over the life cycle are reasonably well approximated by a linear interaction term.

More specifically, Figure 3a shows the estimated next‐period effect of blue‐ versus white‐collar occupations at different ages. The estimated next‐period negative effect of blue‐collar employment is statistically significant from the age of 48 onwards. 12Caution is warranted when interpreting the estimates at the lower and upper ends of the age distribution because of the lower number of observations at young and old ages. Less than 3% of observations occur at ages below 20, and only 2% of observations occur at ages over 60.


Figure 3b‐d refer to estimates obtained from the model in column 6 of Table 3 with added interactions between each of the three occupational stressors and single‐year age dummies. Physical strain has a significant negative effect on workers aged 46 and older. Figure 3c shows consistent negative point estimates for low job control between ages 55 and 61 but with varying precision. The estimated negative effects in Figure 3a‐c are strongest around age 60. Figure 3d shows no evidence of an effect of high psychosocial work load.

Apart from heterogeneity by age, the type of stressors experienced and the vulnerability to certain stressors is likely to differ by gender. Table 4 presents the estimates separately for men and women. The main finding is that the results for men are very similar in sign and significance to the pooled results. The results for women are pretty similar too, although the effect of job control is statistically insignificant and we find no evidence that it increases with age. We are however apprehensive to directly compare the coefficients for women and men for three reasons. First, the levels of the stressors within the same occupation can differ between men and women. Second, women on average have lower physical workload than men (34% of women compared with 43% of men) and so the variation in physical workload among women derives from a different part of the distribution. Third, there is less variation in physical workload among women compared with men and there are fewer observations, leading to lower precision.

**Table 4:** Gender‐specific estimates of the effect of occupational stressors on health

	(1)	(2)	(3)	(4)
**A. Males**				
Blue collar at *t*−1	‐.0481*** (.0196)	.2594*** (.0796)		
Age × Blue collar at *t*−1		‐.0079*** (.0020)		
Physical strain at *t*−1			‐.0767** (.0363)	.4250*** (.1332)
Low job control at *t*−1			‐.0094 (.0541)	.7349*** (.1894)
Psychosocial workload at *t*−1			.0988 (.0598)	‐.0953 (.2018)
Age × Physical strain at *t*−1				‐.0130*** (.0034)
Age × Job control at *t*−1				‐.0186*** (.0046)
Age × Psychosocial workload at *t*−1				.0044 (.0048)
Health at t‐1	.1258*** (.0050)	.1255*** (.0050)	.1257*** (.0050)	.1252*** (.0050)
FE, age and wave dummies	✓	✓	✓	✓
Observations	123,298	123,298	123,298	123,298
**B. Females**				
Blue‐collar at *t*−1	‐.0596** (.0242)	‐.0482 (.0693)		
Age × Blue collar at *t*−1		‐.0003 (.0018)		
Physical strain at *t*−1			‐.1073** (.0441)	.1243 (.1496)
Low job control at *t*−1			‐.0560 (.0643)	‐.1614 (.2696)
Psychosocial workload at *t*−1			.0411 (.0603)	.0850 (.2467)
Age × Physical strain at *t*−1				‐.0060* (.0036)
Age × Job control at *t*−1				.0027 (.0068)
Age × Psychosocial workload at *t*−1				‐.0011 (.0059)
Health at *t*−1	.0950*** (.0055)	.0950*** (.0055)	.0950*** (.0055)	.0950*** (.0055)
FE, age and wave dummies	✓	✓	✓	✓
Observations	99,428	99,428	99,428	99,428

*Notes*. Columns 1 and 2 use the dummy Blue collar as variable of interest (reference category working in a white collar occupation), while columns 3 and 4 use the three occupational stressors as variables of interest. All columns present a specification with fixed effects (FE, deviation from means) and a lagged dependent variable. Standard errors clustered at the occupational title level. * indicates significance at the 10% level, ** at the 5% level, and *** at the 1% level. Intercepts not shown.

### 5.4 Robustness checks

We examine the robustness of our main findings to (a) alternative measures of occupational stressors, (b) using SAH or SF12 instead of HSAT as our outcome measure, (c) investigating time‐varying health shocks, (d) controlling for education‐specific health deterioration by age, (e) limiting the sample to full‐time workers, (f) limiting the sample to individuals who are observed in nine or more waves, (g) applying a correction for health‐related attrition, and (h) two additional ways of specifying our dynamic panel data model. We only present the results for our main specification with age interactions and leave all other results available upon request.

**Measurement of occupational stress.** Table 5 shows that our results are robust to using other measures of occupational stressors and health. Column 1 includes composite measures of physical strain, job control, and psychosocial workload and shows similar results in terms of effect sizes of a standard‐deviation increase in the occupational stressors. We obtain composite measures of the three occupational stressors by averaging percentages of high exposure to (a) physical strain as measured by heavy lifting, working crouched down, and working standing up; (b) job control as measured by work being stipulated in the minutest details, being allowed to plan and schedule work by oneself, and being able to influence the amount of work one has to do; and (c) psychosocial workload as measured by working under great deadline pressure, reaching the limits of one's capacities, and working very quick. Furthermore, analyses using the Finnish job‐exposure matrix instead of the GQCS provide results that are similar in terms of order of magnitude and significance (see Ravesteijn, van Kippersluis & van Doorslaer, 2014).
**Health measures.** SAH—measured on a 5‐point scale—was only included in the SOEP questionnaire in 1992 and from 1994 onwards. Column 2 of Table 5 substitutes SAH for HSAT and the coefficient estimates reveal similar signs and sizes to our main findings in column 6 of Table 3, supporting our conclusion that physical strain and low job control are harmful to health at older ages.
  As additional health measures, we compute the SOEP version of the SF‐12 using six biennial waves between 2002 and 2012 (Andersen, Mühlbacher, Nübling, Schupp & Wagner, 2007). There is a serious concern about the consistency of our dynamic panel data model with only 6 waves, and so our estimates should be interpreted prudently. Nonetheless, it is informative to gauge whether our main findings are robust to using a more comprehensive health measure such as the SF‐12. Columns 3 and 4 show the results for the physical index of SF‐12 and the mental index of SF‐12, respectively. For the measure of physical health, our findings suggest that blue‐collar work and the physical stressor are associated with worse physical health at older ages, in line with our results for health satisfaction. For the measure of mental health, our findings suggest that only the physical stressor is associated with worse mental health at a later age. In contrast to the main results, however, we do not find evidence that job control is associated with worse health in terms of the SF‐12.
**Time‐varying health changes.** We now turn to Table 6 for a second set of robustness checks, now investigating the sensitivity of our results toward changes in the empirical specification. Although the lagged dependent variable captures unobserved time‐varying shocks that decay exponentially, we cannot rule out that other time‐varying shocks influence both occupational switches as well as changes in health. The SOEP data includes information on “the number of hospital days in the past year” and “sickness absenteeism days in the past year.” We include the lag of these variables in our model as proxies for possibly omitted time‐varying health shocks. Column 1 shows that the results are not sensitive to the inclusion of these variables.
**Full‐time workers only.** As an alternative way to gauge the importance of endogenous occupational switches for health reasons, we restrict the sample to individuals who work full‐time in all observed waves. The idea is that occupational switches that lead to a reduction in working hours are more likely to be health induced. Hence, when restricting to full‐time workers, this source of endogeneity should be reduced. Column 2 presents the results restricting to full‐time workers and shows that the results are very similar to our main specification.
**Education‐specific health deterioration by age.** Individuals in different occupations may have different biological ageing rates. We have assumed uniform ageing effects in the preceding analyses. If the health of blue‐collar workers declines more rapidly regardless of their occupation, our results overestimate the harmful effects of physical strain. In column 3 of Table 6, we allow for different rates of ageing by interacting each of the levels of educational attainment with a fifth‐degree age polynomial, and our estimates are similar to our findings in Table 3.
**At least nine waves.** The estimator of the coefficient of the lagged dependent variable is consistent if the number of time periods in the sample goes to infinity. Our sample spans 29 years and is unbalanced because it includes individuals who are observed for fewer waves. We repeat our analysis for a subsample of 10,373 individuals who have been employed for at least 9 of the 29 years to counter the downward bias of the estimator of the lagged dependent variable that plagues short panels (Bond, 2002). The number of person‐wave observations drops from 222,726 in our baseline sample to 151,752 in column 4 of Table 6. The coefficients of the (age‐interacted) occupational stressors are similar to those in our baseline specification. However, the coefficient of lagged health is now larger, suggesting that past health investment, occupational stress, and health shocks are more persistent than they were in the full‐sample analysis. We conclude that our estimates of the effects of occupational stressors are robust across specifications but that an analysis of the full sample leads to underestimation of the coefficient of lagged health, and thereby, we may have underestimated the cumulative effects of occupational history.
**Attrition correction.** Given the long‐running nature of the panel data, clearly attrition due to mortality or nonresponse is a serious issue. Health‐related attrition—if present—will lead to a bias toward zero of our estimators, if individuals with the highest vulnerability to occupation‐related health deterioration are more likely to suffer from attrition. We find that the likelihood of attrition is at most 1% higher for blue‐collar workers than for white‐collar workers in our sample. Moreover, we repeat our analyses using inverse‐probability weights to correct for health‐related attrition on basis of observable characteristics. Specifically, following Jones, Koolman, and Rice (2006), we estimate a Probit model for the propensity to remain in the sample in each wave as a function of the characteristics observed in the first wave an individual is present. These characteristics include health satisfaction, the number of nights in the hospital, the number of days a person is ill, gender, birth year, education, and our occupational characteristics blue collar, physical strain, working under deadline pressure, and job control. We then reestimate our main specification (9), but now using the inverse of the probability that a given individual remains in the sample as sampling weight. The results are presented in column 5 of Table 6 and are very similar to the baseline results. We conclude that attrition bias is unlikely to have a major influence on our conclusions.
**Fixed effects specification.**Angrist and Pischke (2009) have voiced concerns about the violation of strict exogeneity in fixed effects dynamic models, particularly by utilizing short panels. They propose checking robustness by separately estimating both a fixed effects and a lagged dependent variable model. Column 6 of Table 6 presents results from a fixed effects model without a lagged dependent variable. 13With respect to equation 9, the error term would now include the deviations of the effects of health investment, occupational stressors, and health shocks before period *t*−1 from their individual averages. If a past health shock would have a negative effect on current health and lead to higher occupational stress in the previous period, we would overestimate the effect of occupational stressors because this situation leads to additional correlation between **o** and the error term.The point estimates in column 5 suggest a somewhat stronger effect of physical strain and low job control at older ages than the baseline specification. However, these estimates may be the result of a bias caused by past events that affected health and occupational choice that are not accounted for by the lagged dependent variable, which is omitted in this specification.
**Lagged dependent variable specification.** In a model in which we control for a lagged dependent variable, but not for individual‐specific fixed effects, the estimator of the decay parameter *ϕ* in Equation 9 is biased toward one because *h*_*t*−1_ contains *h*_*p*_(see Equation 7), which has a coefficient of one and no longer drops out if we do not subtract $\bar{h}$. We can therefore no longer distinguish between the elements in *h*_*t*−1_ that are transitory and the elements that are constant over time, which explains the bias of the estimator of *ϕ* toward one. In this specification, we therefore overestimate the impact of past events on current health, and we only partly control for unobserved time‐invariant heterogeneity. 14By not subtracting averages in Equation 9, the error term now includes (1−*ϕ*)*h*_*p*_, which may be correlated with lagged health and occupational characteristics.To proxy for time‐invariant unobserved factors otherwise picked up by the fixed effect, we control for years of schooling and gender. Our estimates are now mostly driven by variation between individuals. The coefficients of the interaction between age and occupational stressors in column 7 of Table 6 are similar to our earlier results. Overall, our main conclusions do not change when estimating models that include either individual‐specific fixed effects or a lagged dependent variable, which is reassuring.
  Other methods have been proposed to consistently estimate ***γ***_**o**_ in Equation 9 in short panels, of which the so‐called Arellano–Bond estimator (Arellano & Bover, 1995; Blundell & Bond, 1998) is the most prominent. The Arellano–Bond estimator is based on the first‐difference estimator. The most important assumption is that second and further lags of health are uncorrelated with the first differences of the error term and can be used as instrumental variables for *h*_*t*−1_−*h*_*t*−2_. The Arellano–Bond test for autocorrelation rejects this assumption in our case, which is not surprising because using lagged values as instruments is difficult to justify in the case of health: Chronic illnesses or the introduction of a new medical drug may progressively affect health over time, which leads to second‐ or higher‐order serial correlation in the differenced error term and violation of the exogeneity assumption. In attempting to overcome this problem, more lags of the regressors were included in the model, and further lags of regressors and instruments were used to purge the error term from autocorrelation. However, we still find higher‐order autocorrelation in these models, rejecting the validity of the instruments. 15Limiting the number of waves can give us the false illusion that serial correlation of the error term is not a problem simply because of the low power of the test. Blundell and Bond (1998) and Michaud and van Soest (2008) use short panels of six waves and “use up” even more waves due to the inclusion of lagged values of the dependent variable. The autocorrelation tests in these studies do not reject the assumption of no autocorrelation in the error term, which may be the result of limited test power based on the small number of waves. If we include one‐ and two‐period lags of the dependent variable, we find no second‐order autocorrelation. However, we find autocorrelation of the third‐order, which still violates the Arellano–Bond assumptions. Including third or fourth lags seems to shift the order of autocorrelation downward rather than to solve the problem. The Sargan test may not be informative because it assumes that at least one instrument is exogenous, which is an assumption we are not willing to make.

**Table 5:** Robustness of the measurements for occupational stressors and health

	Composite stressors	SAH	SF12 Physical	SF12 Mental
	(1)	(2)	(3)	(4)
Physical strain at *t*−1	.2689** (.1086)	.1791*** (.0617)	5.8483*** (1.4986)	3.7527** (1.7948)
Job control at *t*−1	.9787*** (.3039)	.2917*** (.0760)	1.6742 (2.0644)	2.2375 (2.6642)
Psychosocial workload at *t*−1	.2375 (.2426)	.0755 (.0816)	‐2.7609 (2.8673)	‐.1922 (2.8271)
Age × Physical strain at *t*−1	‐.0089*** (.0027)	‐.0046*** (.004)	‐.1296*** (.0347)	‐.1012** (.0424)
Age × Job control at *t*−1	‐.0279*** (.0079)	‐.0059*** (.0019)	‐.0474 (.0495)	‐.0430 (.0620)
Age × Psychosocial workload at *t*−1	.0044 (.0058)	‐.0027 (.0022)	.0586 (.0655)	.0178 (.0655)
Health at *t*−1	.1116*** (.0039)	.0722*** (.0036)	‐.1426** (.0088)	‐.1475*** (.0073)
Age and wave dummies	✓	✓	✓	✓
Observations	222,726	162,595	39,599	39,599

*Notes*. Robustness checks for measurement. Standard errors clustered at the occupational title level. * indicates significance at the 10% level, ** at the 5% level, and *** at the 1% level. Intercepts not shown. Columns 3 and 4 are estimated on six biennial waves between 2002 and 2012. Source: SOEP v29, GQCS.

**Table 6:** Robustness of the empirical specification

	Time‐varying shocks	Full‐time workers	Education‐specific age	*T*>8	Attrition correction	FE	LDV
	(1)	(2)	(3)	(4)	(5)	(6)	(7)
Physical strain at *t*−1	.4213*** (.1195)	.5640*** (.1890)	.4086*** (.1153)	.3467*** (.1138)	.6259*** (.1374)	.4696*** (.1206)	.1310** (.0568)
Job control at *t*−1	.3364*** (.1681)	.6558** (.2784)	.3577** (.1635)	.4435** (.1767)	.4530** (.2094)	.4048** (.1691)	.3959*** (.1107)
Psychosocial workload at *t*−1	.0928 (.1884)	.0916 (.3200)	.1052 (.1630)	.4229** (.1767)	‐.1124 (.2553)	.1536 (.1777)	.1315 (.1103)
Age × Physical strain at *t*−1	‐.0129*** (.0029)	‐.0135*** (.0044)	‐.0128*** (.0029)	‐.0155*** (.0033)	‐.0127*** (.0031)	‐.0145*** (.0031)	‐.007*** (.0014)
Age × Job control at *t*−1	‐.0092** (.0042)	‐.0161** (.0065)	‐.0095** (.0041)	‐.0111** (.0043)	‐.0110** (.0051)	‐.0109** (.0043)	‐.0131*** (.0027)
Age × Psychosocial workload at *t*−1	‐.0011 (.0045)	‐.0028 (.0074)	.0011 (.0039)	‐.0073 (.0048)	.0023 (.0060)	‐.0019 (.0043)	.0035 (.0027)
Health at *t*−1	0.1061*** (0.0041)	.0546 (.0051)	.1113*** (.0039)	.1612*** (.0040)	.0659*** (.0054)		.5464*** (.0043)
Age and wave dummies	✓	✓	✓	✓	✓	✓	✓
Age dummies interacted with education dummies	✗	✗	✓	✗	✗	✗	✗
Individual FE	✓	✓	✓	✓	✓	✓	✗
Education and gender	✗	✗	✗	✗	✗	✗	✓
Hospital days and sickness absenteeism	✓	✗	✗	✗	✗	✗	✗
Observations	207,073	89,083	222,726	151,752	222,726	222,726	222,726

*Notes*. FE refers to fixed effects estimation (deviation from means), and LDV refers to the inclusion of the lagged dependent variable. Standard errors clustered at the occupational title level. * indicates significance at the 10% level, ** at the 5% level, and *** at the 1% level. Intercepts not shown. Source: SOEP v29, GQCS.

## 6 CONCLUSION

The strong association between occupation and health is widely documented. Our results confirm that German blue‐collar workers report worse health than white‐collar workers, and that the size of this health gap is comparable to the effect of ageing 29 months. However, because of various sources of selection into occupation, the association does not necessarily reflect the causal effect of occupation on health.

Identification of health effects of occupations is policy relevant for at least two reasons. From a fairness perspective, one could be more concerned by health inequalities that derive from highly restricted choice sets, such as occupational choices which are restricted by endowments, in comparison to more freely chosen health behaviors. From a productivity perspective, it is vital for both regulators and employers to know which specific occupational characteristics are most harmful to health. For example, occupations with harmful ergonomic workplace conditions may simultaneously be characterized by low control possibilities at work, which may exert an independent effect on health. Consequently, separating out these effects is crucial for better‐targeted efforts at reducing sickness absenteeism and disability by adjusting specific labor conditions.

In this paper, we make three contributions. First, by proposing a dynamic theoretical model as the foundation of our empirical specification, we highlight the various sources of selection into occupation, and we make explicit under which conditions the coefficient of occupational stressors can be interpreted as causal.

Second, we estimate the empirical equation deriving from the theory using a detailed German longitudinal dataset over many time periods (29 years). In this equation, we account for the sources of selection: (a) unobserved time‐invariant variables due to the inclusion of individual fixed effects, (b) time‐varying observed variables such as age and wave dummies, and (c) time‐varying unobserved shocks that exponentially die out through the inclusion of the lagged dependent variable. In doing so, we are able to quantify a lower bound on the selection effect, and an upper bound on the causal effect of occupation on health. Moreover, our results generalize across the entire labor force, which is in contrast to local effect estimates based on a particular reform that affected only part of the employed population.

Third, we go beyond the literature by estimating separate health effects of physical strain and Karasek's two dimensions of psychosocial occupational stress: job control and psychosocial workload from the GQCS.

Our main finding is that selection of individuals with poor health (prospects) into occupations with heavy physical demands and low job control, accounts for at least 60% of the observed association with health. The association between psychosocial workload and health seems to be entirely driven by selection. Although we cannot rule out other sources of selection, if we interpret the conditional differences in health as deriving from the occupational characteristics, we find that both high physical occupational demands and low job control have negative effects on health and that these effects increase with age. The effect of exposure to a one‐standard‐deviation increase in the degree of handling heavy burdens (e.g., working as a toolmaker instead of a teacher) between ages 50 and 54 is comparable to ageing 16 months. Low job control is harmful to health but only after age 48. The effect of exposure to a one‐standard‐deviation decrease in the degree of job control (e.g., working as a secretary instead of a librarian) between ages 50 and 54 is comparable to ageing 6 months.

Because we do not observe all individual levels of health investment, we are unable to disentangle the effects of occupational stressors and any health investment made in response to occupational choice. Even if we would observe all health investments, we could not directly include those behavioral adjustments to our specification due to the “bad control problem.” Nonetheless, we have investigated whether health investments such as smoking and sports activity are outcomes of occupational stressors (results available upon request). The effects of occupational stressors on health investments are limited. If anything it seems that blue‐collar work and heavy physical strain lead to lower health investments at older ages. This implies that we may slightly overestimate the structural effect of occupational stressors, ***γ***_**o**_, in Equation 2. Still, we believe the composite coefficient we estimate is a policy‐relevant effect, since it can be interpreted as the sum of the direct effect of occupation and the indirect effect of any behavioral response to occupational choice.

Occupational health and safety policies, career development programs, and retirement policies should be based on the knowledge that selection into occupation explains a substantial fraction of health disparities across occupational groups. It is important to emphasize that ignoring this selection will lead to serious overestimation of the benefits of improving occupational characteristics. Our results suggest that exposure to physical strain and low job control is harmful to health at older ages. That would mean that successfully shielding workers—especially older workers—from such conditions will prevent accelerated health deterioration and therefore also illness‐related absenteeism and labor force exit due to work disability. Our findings therefore have great relevance for the many OECD countries that are aiming to extend working life careers in order to keep disability and pension systems sustainable.
